# Health Information Adoption Among Patients With Chronic Disease in China: Qualitative Interview Study of Patient–Platform Coshaping

**DOI:** 10.2196/85229

**Published:** 2026-05-20

**Authors:** Lin Huang, Guimin Duan, Jiani Tang, Peipei He

**Affiliations:** 1 School of Management, Chengdu University of Traditional Chinese Medicine, 1166 Liutai Avenue, Wenjiang District, Chengdu, Sichuan, 611137, China, 86 13568881021; 2 School of Public Administration, Sichuan University, Chengdu, Sichuan, China

**Keywords:** digital health information, digital platforms, information adoption, chronic diseases, grounded theory

## Abstract

**Background:**

Chronic disease management increasingly relies on digital health information. Traditional adoption models conceptualize adoption as an individual decision determined by cognitive evaluations. However, in contemporary platform environments, health information exposure and interaction are shaped by algorithmic curation and platform governance. Understanding how patients with chronic disease engage with these platform-mediated spaces requires examining adoption as an ongoing process rather than a discrete outcome. Such process-oriented understanding remains limited.

**Objective:**

This study aims to construct a theoretical model that captures the dynamic, processual nature of health information adoption among patients with chronic disease in platform-mediated environments, with particular attention to the reciprocal interactions between patient practices and platform structures.

**Methods:**

This study used grounded theory methodology and conducted in-depth semistructured interviews with 32 patients with chronic disease in Chengdu, Sichuan, China, from December 2023 to July 2025. Participants included individuals with various chronic conditions such as hypertension, rhinitis, lumbar disc herniation, urticaria, and other persistent health conditions requiring sustained self-management. The sample was diverse in age, gender, and educational background. Theoretical sampling was used across 3 stages to ensure diversity and category saturation. Data were systematically analyzed through 3 levels of coding: open coding generated 22 categories, axial coding organized these into 7 major categories, and selective coding integrated these into 3 core categories. Particular attention was paid to how patients interpreted, evaluated, and integrated digital health information through continuous interaction with platform technologies and governance structures.

**Results:**

Analysis identified 3 core categories of health information adoption—adoption propensity, platform context, and intervening conditions—which were integrated into a process model. Adoption Propensity comprises 3 interconnected layers, including motivational (disease learning, information verification, and comfort seeking), behavioral (information seeking, analysis, keeping, and adoption), and attitudinal (cognitive, emotional, and contextual resonance). Platform Context includes both information context (information content, information format, and information source) and institutional context (platform technology, platform characteristics, and platform governance). Intervening Conditions consist of individual circumstances (foundational characteristics, information literacy, and illness experience) and environmental conditions (emergency situations, policy changes, and social relationships). The analysis further revealed a cyclical co-construction process, in which patient behaviors and platform environments continuously shape each other through reciprocal interactions.

**Conclusions:**

This study proposes a theoretical model that explores how the interaction between digital platforms and patients influences health information adoption. By adopting an interpretive approach and introducing the patient-platform coconstruction perspective, this research extends existing health information behavior theories to account for platform-era dynamics. The model highlights the crucial role of platforms in structuring patients’ ongoing health information practices, providing insights for the design, governance, and optimization of digital health platforms to better support chronic disease self-management.

## Introduction

The rapid expansion of digital health technologies has fundamentally transformed how individuals access, evaluate, and adopt health information [1,2]. Digital health information adoption has become a routine yet consequential information behavior [3], directly influencing health decision-making and long-term health outcomes. Digital platforms now serve as the primary infrastructures through which individuals encounter and make sense of health information. In the Chinese context examined in this study, the public relies on professional health platforms such as Dingxiangyuan and the Health China app, alongside mainstream social media platforms including Weibo, WeChat official accounts, Douyin, and Kuaishou. Together, these platforms constitute an integrated digital health information ecosystem characterized by algorithmic recommendation systems, interactive affordances, content moderation practices, and commercially driven governance logics.

However, the growing dominance of these platforms and their algorithmic systems has profoundly restructured health information environments, altering not only what information users encounter but also how they engage with it. Empirical studies indicate that misinformation, uneven information quality, algorithmic amplification, and insufficient platform governance increasingly complicate individuals’ ability to adopt accurate and relevant health information [4-6]. Under these conditions, digital health information adoption no longer occurs within a stable and neutral information environment; instead, it unfolds within continuously mediated, algorithmically curated, and commercially governed platform ecosystems.

Existing theoretical frameworks of digital health information adoption, including the information adoption model, technology acceptance model, unified theory of acceptance and use of technology, theory of planned behavior, and the elaboration likelihood model, have substantially advanced understanding of the attitudinal, cognitive, and social predictors of adoption intentions [7-14]. These frameworks, however, have predominantly been operationalized within post-positivist paradigms that conceptualize adoption as a discrete behavioral outcome predicted by measurable variables. Their central concern is identifying which factors determine whether information will be adopted at a specific decision point. While analytically rigorous, this outcome-oriented orientation treats the information environment as a relatively stable external backdrop. As a result, these models are limited in their capacity to explain how adoption unfolds as an ongoing, experience-based process embedded in dynamic platform environments characterized by algorithmic mediation, passive exposure, fragmented information consumption, and evolving governance interventions.

Conceptually, this limitation reflects a deeper epistemological tension. Variable-centered adoption models prioritize prediction and causal explanation, whereas contemporary platform environments require interpretive understanding of how individuals make sense of information under conditions shaped by algorithmic visibility, social signals, and governance structures. In platform-mediated contexts, health information adoption unfolds through iterative interactions among users, health information, and platform architectures. Accordingly, this study adopts an interpretive epistemological stance, conceptualizing digital health information adoption not as a singular outcome but as a dynamic process of meaning-making and negotiation within sociotechnical systems.

Understanding how these platform-mediated environments structure health information adoption requires examining the technological infrastructures and governance mechanisms that shape information flows and user conduct. Scholarship on platform governance provides a critical lens for this examination. Robert Gorwa conceptualizes platform governance as the combination of technical architectures, institutional rules, and market incentives through which platforms regulate participation and visibility [15]. Related scholarship demonstrates that community standards and content moderation practices operate as quasi-regulatory mechanisms shaping information exchange [16], and that algorithmic systems systematically influence exposure patterns, credibility cues, and behavioral feedback loops [17-25]. In health information contexts, algorithmic curation blends personalization, social endorsement, and regulatory intervention, thereby actively configuring what users see, trust, and consider actionable. Importantly, platform structures do not merely serve as passive channels for information delivery; they constitute active sociotechnical environments that both shape and are reshaped by user practices. While platform governance scholarship provides crucial insights into how digital infrastructures shape information environments, its integration into health information adoption research remains limited, particularly in understanding how patients navigate and respond to these governed spaces.

Within these platform-mediated environments, patients with chronic disease constitute a particularly critical population for examining digital health information adoption. In this study, patients with chronic disease refer to individuals living with long-term health conditions requiring sustained self-management and ongoing information engagement [26], including but not limited to cardiovascular diseases, diabetes, chronic respiratory conditions, chronic musculoskeletal disorders, endocrine disorders, and other persistent health conditions. Chronic diseases account for more than 70% of global mortality and impose substantial and sustained burdens on health systems worldwide [26]. Beyond clinical care, chronic disease management entails continuous decision-making related to medication adherence, lifestyle adjustment, symptom monitoring, and psychological regulation. Prior research conceptualizes patients with chronic disease as highly information-dependent self-managers whose health behaviors are iterative, context-sensitive, and shaped by ongoing information engagement [2]. Their health outcomes are therefore deeply intertwined with how they access, interpret, evaluate, and ultimately adopt digital health information over time.

Despite growing research attention to digital health information behaviors, 3 interrelated gaps remain. First, although health information adoption has been examined across diverse populations, comparatively limited focused attention has been paid to patients with chronic disease, particularly those whose information engagement is sustained, iterative, and consequential [27]. Second, dominant adoption frameworks emphasize predictive determinants of behavioral intention while insufficiently accounting for the processual, experience-based, and contextually embedded nature of adoption in algorithmically mediated environments [7-14]. Third, existing research rarely integrates platform perspectives into qualitatively grounded analyses of health information adoption, leaving underexplored the reciprocal dynamics through which patients’ information practices interact with and respond to platform architectures and regulatory mechanisms.

Addressing these gaps requires reconceptualizing digital health information adoption as a platform-embedded process shaped by continuous interactions among individual sensemaking, informational characteristics, and platform mechanisms such as algorithmic curation and governance structures. Guided by grounded theory and informed by an interpretive research orientation, this study seeks to develop a process-oriented theoretical account of how patients with chronic disease adopt digital health information within platform-mediated environments. Specifically, this study addresses the following research questions: (1) What key components characterize patients with chronic disease’s health information adoption behaviors? (2) How do platform technologies, algorithmic recommendations, and governance mechanisms feature in patients with chronic disease’s health information adoption experiences? (3) How can a process-oriented theoretical model be developed to explain the dynamics of health information adoption among chronic disease patients in platform-mediated contexts? By theorizing adoption as an iterative, relational, and platform-embedded process, this study aims to contribute to health information behavior scholarship and to inform the design and governance of digital health platforms in ways that better support sustainable chronic disease self-management.

## Methods

### Overview

Grounded theory represents a qualitative research methodology embedded within an interpretivist paradigm, wherein the social world is understood as constructed through participants’ lived experiences and subjective meanings [28]. Its fundamental objective is to construct a theory from empirical data [29]. Researchers conduct investigations without preconceived theoretical assumptions, engaging in comprehensive and in-depth exploration of phenomena within their natural contexts. Through continuous inductive processes and data condensation, researchers derive categories from raw data, ultimately formulating theoretical frameworks. This study uses a combination of face-to-face and telephone interviews to gather primary data from patients with chronic disease. Data organization and coding are conducted through 3 analytical levels using NVivo (version 14; QSR International) software, accompanied by theoretical saturation testing to ensure the authenticity and reliability of research findings. This study followed the SRQR (Standards for Reporting Qualitative Research) guideline (Checklist 1) [30].

### Study Setting and Participants

#### Theoretical Sampling Strategy

In grounded theory, data saturation serves as the core criterion for determining sample size adequacy. It refers to the stage where, after continuous sampling and data analysis, new data no longer provide novel theoretical insights or categories [29]. At this stage, the sample size is considered saturated, and sampling can be discontinued. This study avoids pre-establishing fixed sample sizes, instead adopting a data-driven approach through continuous coding, theoretical sampling, and saturation testing to ensure the sufficiency and accuracy of theory construction [31].

It is important to clarify that this study used theoretical sampling, not random sampling. Theoretical sampling is a hallmark of grounded theory methodology, wherein sampling decisions are guided by emerging concepts and categories rather than predetermined criteria. Initial participants were selected purposively to ensure diversity in characteristics such as age, disease type, and digital literacy. As analysis progressed, subsequent participants were selected based on their ability to provide insights into emerging categories and to test the boundaries of developing concepts.

#### Recruitment Setting and Procedures

This study was conducted in urban Chengdu, China, selecting patients with chronic disease from multiple districts, including Wenjiang, Wuhou, Jinjiang, and Shuangliu, for community-based investigation. Recruitment was conducted through multiple channels to ensure diversity: (1) community health centers: Researchers collaborated with community health centers to identify potential participants during routine health checkups and chronic disease management programs, (2) patient support groups: Online and offline communities of patients with chronic disease were contacted to recruit participants, (3) Snowball sampling: Initial participants were asked to refer other patients with chronic disease who might be interested in participating.

The following outlines the specific sample confirmation process across 3 sequential stages:

- Stage 1—Initial Sampling (n=10): Initial participants were selected based on research objectives, with inclusion criteria as follows: (1) Clear understanding of digital health information needs described in the interview protocol, particularly focusing on their perspectives regarding the relevance of digital health information in daily health management; (2) At least 18 years of age, possessing cognitive abilities for coherent expression of experiences and viewpoints, providing rich data for grounded theory analysis; (3) Signed written informed consent, agreeing to interview recording and anonymous data usage while retaining the right to withdraw at any time; (4) Capability to participate in 15‐50 minute semistructured interviews for in-depth exploration of their digital health information adoption journeys.

Through the first stage of interviews and initial open coding, 3 preliminary categories emerged: digital health information adoption motivation, digital health information adoption behavior, and digital health information adoption attitude.

- Stage 2—theoretical sampling for diversity (n=5): To maximize category heterogeneity and explore the range of experiences, we recruited diverse samples based on factors such as age, education level, and occupation. This stage specifically targeted older individuals, retirees, and hospital administrators to capture perspectives across different demographic and professional backgrounds. The second stage of interviews revealed new categories, indicating that platform information environments and institutional environments influence patients’ digital health information behaviors.
- Stage 3—targeted theoretical sampling (n=15): Consequently, this study supplemented samples to further explore platform-related phenomena. In addition to meeting initial inclusion criteria, supplementary respondents in this stage were required to be patients with chronic disease with certain digital literacy levels, capable of articulating their experiences with various digital platforms and platform features. This targeted sampling allowed for deeper exploration of how platform algorithms, governance, and design influence information behaviors.
- Saturation testing (n=2): In stage 3, no new codes or categories emerged during the coding of the 15th interview sample, suggesting that theoretical saturation had been reached. To verify this, 2 additional samples were collected and analyzed; again, no new codes or categories appeared, confirming the stability of the existing categories and indicating that no further sampling was necessary.

Adopting an iterative approach, this study follows the dynamic sampling framework of grounded theory. This approach focuses on achieving categorical depth by using a heterogeneous sample. The sample size is thus guided by the sufficiency of information, not statistical considerations [29]. In addition, previous studies have successfully used grounded theory for research of moderate complexity, with their sample sizes varying from 18 to 30 [5]. Based on these considerations, the final sample size of 32 participants for this study is deemed appropriate.

### Data Collection

In this study, prior to conducting in-depth field interviews, we developed an interview protocol encompassing multiple dimensions, including topics of concern to patients with chronic disease, demand levels, health information behavior processes, and digital platform usage experiences (Table 1).

**Table 1.** Interview guide on digital health information adoption by patients with chronic disease.

Category	Interview questions
Basic information	Q1: Age Q2: Sex assigned at birth Q3: Educational background Q4: Occupation Q5: Do you have any chronic illnesses? if so, what are they?
Question Design	Q6：Do you typically follow health-related information? If so, what specific topics or aspects of health information do you focus on? Q7: In your daily life, how important is health information to you? Why? Q8: Could you please tell us how you began to seek out and engage with health information after being diagnosed with a chronic illness? Q9: What channels do you typically use to get health information? How do you evaluate the reliability of that information? And how satisfied are you with its quality and relevance? Q10: Could you provide an example of how you searched for a specific piece of health information and then put it into practice? What challenges did you face during that process? Q11: What formats (eg, text, video, infographics) are most common in the health information you follow? Which format do you prefer, and why? Do you have any suggestions for improving how this information is presented? Q12: How helpful has the health information you’ve adopted been for your health condition or lifestyle? Q13: What are your general impressions or feelings about the platforms and individuals (eg, doctors, apps, bloggers) that provide this health information? Q14: To conclude, do you have any other experiences or thoughts about adopting health information that you’d like to share?

Prior to conducting the formal interview, this study conducted rigorous pretesting of the interview protocol. During testing, research team members alternately assumed roles of interviewer and interviewee, simulating authentic interview scenarios. They evaluated the protocol from perspectives of question clarity, logical coherence, and guidance. Based on pretest feedback, adjustments were made to ambiguously worded questions, question sequences were optimized, and more targeted follow-up statements were added to enhance interview validity and reliability. This pretesting phase laid a solid foundation for the smooth implementation of the formal interviews and helped ensure the quality of the research data.

Data collection and analysis proceeded simultaneously and were closely integrated, following grounded theory principles. Semistructured interviews were conducted from December 2023 to July 2025. Following the interview protocol (Table 1), each interview lasted approximately 15 to 50 minutes. Interviews were conducted either face-to-face or by telephone, according to participants’ preferences and practical circumstances. This mixed-mode approach was adopted for two reasons: (1) to accommodate participants with mobility limitations due to their health conditions, thereby enhancing accessibility and inclusivity; and (2) to respect participants’ comfort levels and scheduling constraints, as some patients with chronic disease preferred the convenience of telephone interviews due to time availability or personal preference. Prior research has demonstrated that telephone and face-to-face interviews yield comparable data quality in qualitative health research when using semistructured protocols [32]. In our study, no systematic differences in interview depth or quality were observed between the 2 modes. Interview settings were determined by the participants and included private or semiprivate locations such as their homes, cafés, or university office buildings, with no individuals other than the participant and the interviewer present.

The interviews were conducted by the first author, who, during the informed consent process, disclosed her personal experience as a user of digital health information. This appropriate self-disclosure was intended to build trust, reduce power imbalances, and allow participants to comfortably share their genuine experiences. Throughout the interviews, the interviewer maintained strict neutrality, using standardized prompts to avoid influencing the participants’ responses. To minimize bias, the research team pretested the interview questions, ensuring that the self-disclosure statements did not contain leading assumptions. Additionally, a 2-step review process was implemented: recordings were reviewed, and verbatim transcripts were independently cross-checked by other authors to avoid inadvertently suggesting answers based on personal experience. These authors were further responsible for ensuring that all issues concerning the accuracy and completeness of the research were properly addressed.

The interview consists of 4 steps. First, the researcher introduces the study and obtains informed consent, giving the participant an opportunity to ask questions or choose to withdraw. This is followed by the question-and-answer session. During interviews, any ambiguities were immediately addressed by requesting clarification from respondents. Furthermore, new questions emerging during interviews were pursued through follow-up questioning until no additional questions arose. After the Q&A, the interviewer summarizes the key discussion points and verifies the accuracy of the information with the participant, inviting them to add any details that may have been overlooked or were incomplete. This step is designed to ensure the comprehensiveness and accuracy of the data collected. Finally, the interviewer thanks the participant for their time and contribution, explains the subsequent processing of the interview data, and assures the participant of the confidentiality of their personal information and responses.

All interviews were conducted with participants’ informed consent and were audio-recorded when permission was granted. Audio recordings were transcribed verbatim using a combination of transcription tools, including iFLYTEK and Apple Voice Memos, and were transcribed as soon as possible after each interview. The first author manually checked all transcripts against the original audio recordings to ensure accuracy. Sixteen participants declined audio recording; in these cases, detailed contemporaneous handwritten notes were taken during the interviews and expanded into full interview records immediately afterward. Interviews were conducted in Mandarin Chinese and the Sichuan dialect. All transcripts and interview records were maintained in Chinese for coding and analysis to preserve linguistic nuances. Representative quotations were subsequently translated into English for publication by a bilingual researcher and independently verified by another bilingual team member.

### Data Analysis

Data analysis followed grounded theory principles and was conducted iteratively and concurrently with data collection [33]. Following Strauss and Corbin’s approach, analysis proceeded through 3 levels of coding: open coding, axial coding, and selective coding. A constant comparative approach was used throughout all coding stages to facilitate theory generation, involving continuous comparison across interview data, codes, and emerging categories. NVivo 14 qualitative data analysis software was used to support data management, coding, and retrieval.

**Figure 1.**
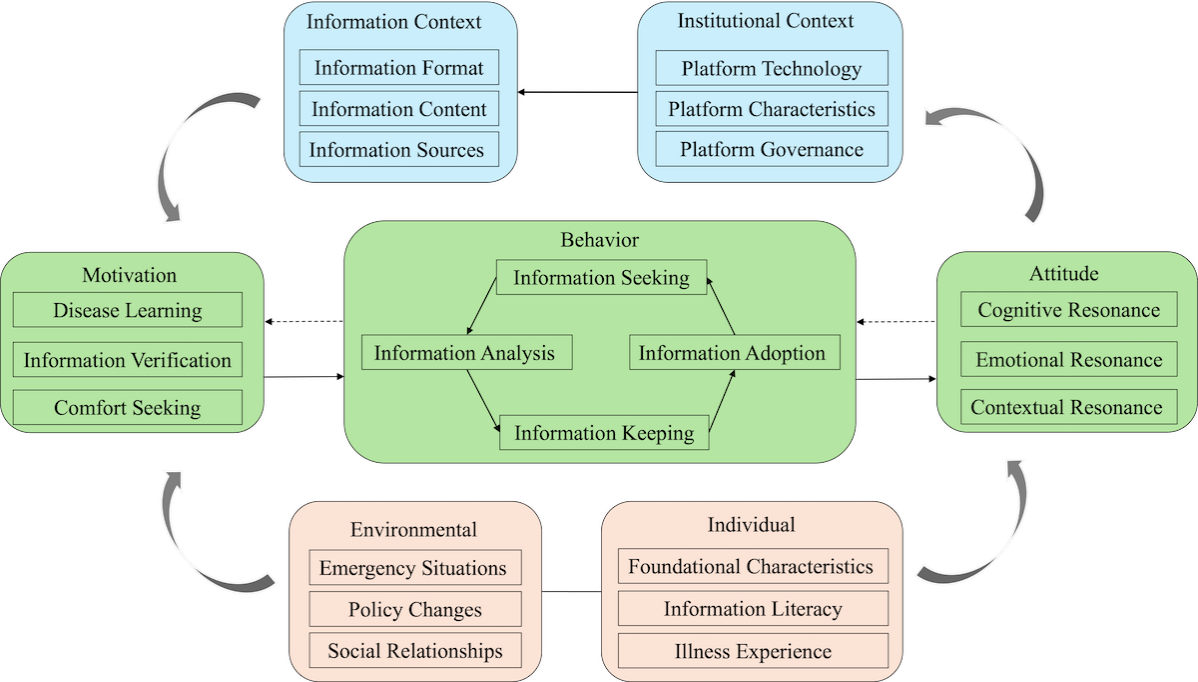
A process model of digital health information adoption by patients with chronic disease.

The 3 levels of coding were operationalized as follows:

- Open coding: Interview transcripts were analyzed line by line to identify meaningful units and inductively generate initial concepts from participants’ statements. Each segment of text was examined to identify distinct phenomena, actions, or experiences. For example, when participants described “searching for disease information on platforms,” “following health bloggers,” and “clicking on algorithm-recommended health videos,” these distinct concepts were identified and subsequently grouped into the category “Information Seeking.” Through iterative team discussions and constant comparison, researchers refined, merged, and organized these concepts, resulting in 22 categories (eg, information seeking, information analysis, illness experience, platform technology, cognitive resonance).
- Axial coding: The 22 categories from open coding were organized into broader major categories based on their logical relationships and conceptual connections. This stage focused on identifying patterns and grouping related categories. Through this process, 7 major categories emerged: individual circumstances, environmental conditions, motivation, behavior, attitudes, information context, and institutional context. For instance, categories related to “information seeking,” “information analysis,” “information keeping,” and “information adoption” were grouped under the major category “behavior.”
- Selective coding: The 7 major categories were systematically integrated to identify core categories that could connect the entire theoretical framework. This stage involved identifying the central phenomenon of digital health information adoption by patients with chronic disease and determining how the major categories related to it. Through this integration process, 3 core categories emerged: (1) Adoption Propensity (integrating motivation, behavior, and attitudes); (2) Platform Context (integrating information context and institutional context); and (3) Intervening Conditions (integrating individual circumstances and environmental conditions). Importantly, selective coding also revealed the dynamic interrelationships among these core categories, particularly the cyclical coconstruction process through which patient behaviors continuously shape and are reshaped by the platform. These core categories and their interrelationships formed the basis of the process model presented in Figure 1. A detailed overview of the coding process, including all categories and their definitions, is provided in the Supplemental Codebook.

To enhance analytic rigor and reduce subjective bias, multiple strategies were used throughout the analysis. The first author conducted initial coding independently, while 2 additional researchers with formal training in qualitative methods independently coded a subset of transcripts (6/32, 18%). Coding discrepancies were discussed during regular team meetings, during which the coding framework was iteratively refined until consensus was reached. Researchers with complementary disciplinary backgrounds, including information science and clinical medicine, contributed to these discussions by critically reflecting on the clarity, coherence, and applicability of participant-derived codes, without introducing externally imposed categories.

The researcher’s reflexivity was integrated throughout data collection and analysis. Reflexive notes were written after each interview and coding session to document researchers’ assumptions, reactions, and evolving interpretations. Contextual reflexivity was maintained by examining how participants’ health conditions, information environments, and situational contexts shaped their accounts. Relational reflexivity was addressed by continuously assessing the distinction between researchers’ perspectives and participants’ meanings. To mitigate potential disciplinary bias, the interview guide was collaboratively developed and pilot-tested, interviewer self-disclosure was intentionally limited, and interview transcripts were reviewed by coauthors who had not conducted the interviews to identify potential leading questions or biased prompts.

Theoretical saturation was assessed iteratively throughout the analysis. Initial coding began after the first stage of interviews (n=10), with subsequent interview stages used to refine categories and theoretical relationships. During coding of the 30th interview, no new codes or categories emerged; therefore, 2 additional interviews were conducted and analyzed using the same coding procedures to verify theoretical saturation. As no new codes, categories, or relationships emerged, theoretical saturation was considered to have been achieved.

To further enhance analytic credibility, expert validation was conducted after the coding and theoretical model development were completed, based entirely on participant data. The preliminary model and analytic interpretations were presented to a panel of 5 experts, including 2 health informaticians, 2 information behavior researchers, and 1 specialist in chronic disease management. Experts were invited to assess the logical coherence of model components and relationships, identify potential gaps or inconsistencies, and comment on the theoretical contribution and practical relevance of the model. Expert feedback was used to refine the clarity and articulation of the model and its implications, without introducing new categories or altering the core relationships derived from participants’ accounts. This process functioned as an additional analytic validation strategy to strengthen conceptual coherence while preserving the participant-driven nature of the grounded theory analysis.

### Ethical Considerations

This study was approved by the Institutional Ethics Review Committee of the School of Public Administration at Sichuan University (approval number 202512300007). All procedures involving human participants adhered to institutional ethical guidelines for social science research. Written informed consent was obtained from all participants prior to data collection. Participants were informed of the purpose of the study, research procedures, potential risks and benefits, the voluntary nature of participation, and their right to refuse or withdraw at any time without negative consequences. To protect privacy and confidentiality, all interview data were anonymized during transcription and were stored on encrypted, password-protected servers accessible only to the research team. Identifiable information such as names, contact information, and specific workplace or residential identifiers was removed. Quotations used for publication were deidentified, and pseudonyms were assigned. No financial or material compensation was provided to participants due to the minimal-risk nature of the study and in accordance with institutional ethics guidelines. This study involved minimal risk and did not include any medical intervention or clinical experimentation.

## Results

### Summary

A total of 32 adult patients with chronic disease participated in our interviews, with no dropouts during the process. Participants represented a variety of chronic conditions, including but not limited to diabetes, hypertension, thyroid disorders, chronic respiratory diseases, and musculoskeletal conditions, with disease duration ranging from 1 to 15 years (mean 6.3, SD 4.2 years). All participants regularly used digital platforms to obtain health information, primarily social media platforms (eg, WeChat, Douyin) and professional health platforms (eg, DingXiangYuan.cn). Table 2 summarizes the sociodemographic characteristics of the sampled participants. This detailed participant information provides context for their health information adoption behaviors and enables assessment of the transferability of the findings.

**Table 2.** Participant demographics (n=32).

Norm and variable	Value, n (%)
Sex assigned at birth	
Male	8 (25)
Female	24 (75)
Age (years)	
18‐44	18 (56)
45‐59	9 (28)
≥60	5 (16)
Educational background	
Primary school	5 (16)
Middle school	4 (13)
High school	3 (9)
College	20 (62)
Occupation	
Professional or technical worker	10 (31)
Service worker	4 (13)
Educator	1 (3)
Self-employed	3 (9)
Farmer	2 (6)
Student	8 (25)
Retiree	4 (13)

The sample comprised predominantly female participants (24/32, 75%). The age distribution covered young adults to seniors, with the majority (18/32, 56%) in the 18‐44 age range. Educational attainment was relatively high, with 62% (n=20) having college-level education, reflecting the urban setting of Chengdu and the digital literacy requirements for engaging with web-based health platforms. Occupational diversity was achieved, with participants’ occupations ranging from farmers to professionals.

Through in-depth interviews and grounded theory analysis with patients with chronic disease, this study seeks to explore their health information adoption behaviors within contemporary digital environments. Research findings reveal that patients’ health information behaviors do not constitute a linear, rational, individual adoption process, but rather represent a complex, dynamically cyclical process characterized by bidirectional interaction with platform information, technology, and governance mechanisms. Specifically, motivation-behavior-attitude constitutes patients’ 3-tiered adoption propensity, while platform Information Contexts and Institutional Contexts serve as the operational domains for patients’ health information behaviors. The unfolding of this cyclical process is further shaped by patients’ individual circumstances and broader socioenvironmental conditions. Based on these findings, this study constructs a process model for patients with chronic disease’s digital health information adoption (Figure 1).

### Core Categories of Digital Health Information Adoption

This study develops a grounded theoretical model of digital health information adoption, comprising 3 core categories: adoption propensity, platform context, and intervening conditions.

#### Adoption Propensity

In patients’ health information adoption processes, “motivation-behavior-attitude” constitutes a dynamic and interconnected, comprehensive explanatory framework.

##### Motivational

The motivational layer represents the starting point of patients’ health information adoption, addressing the question of “why patients actively or passively engage with health information.” Patients with chronic disease are driven by 3 primary motivations: disease learning, information verification, and comfort seeking.

Disease learning represents a central and ongoing motivation in patients with chronic disease’s engagement with health information. This motivation often becomes particularly salient at the time of diagnosis, when patients encounter urgent knowledge gaps and seek to understand their condition. One participant diagnosed with urticaria described this immediate drive:

*It came so suddenly, I wanted to know what caused it, so I searched for a lot of information and confirmed I was allergic.*

[P2]

This account illustrates how the abruptness of a chronic diagnosis can rapidly transform a person into an active, high-intensity information seeker. Another participant, a patient with thyroid cancer who has practiced self-management for 5 years, captured the pervasiveness of this motivation more broadly:

*Nowadays everything is online. You can find whatever you're looking for and use it to learn and get information.*

[P7]

Together, these accounts illustrate how the onset of chronic illness often prompts an immediate turn to digital platforms as key sources for understanding symptoms, causes, and disease management.

Beyond disease learning, patients are also motivated by a need to verify the health information they encounter, particularly as they grow more experienced with the volume and variability of online content. As one older participant with hypertension expressed:

*I have been diagnosed for many years and have always prioritized health preservation, but I still feel there should be content teaching us how to spot fake information—for example, can eating less salt really cure high blood pressure?*

[P10]

This account reflects a meta-cognitive awareness of misinformation risk that emerges through repeated platform engagement, suggesting that information verification motivation intensifies as patients accumulate digital health literacy over time. Another participant with thyroid nodules shared the following experience:

*Once I came across an article saying that ‘people with thyroid nodules shouldn’t eat cruciferous vegetables.’ It scared me so much that I didn’t even dare to eat broccoli. Later, an official clarification debunked the claim and I learned that eating them cooked doesn’t have much impact. I hope public accounts can indicate their information sources and stop scaring people.*

[P13]

A third and often underappreciated motivation is comfort seeking—the need to regulate negative emotions generated by chronic illness. Chronic disease imposes persistent uncertainty, physical discomfort, and psychological burden, and patients actively seek out information that can alleviate anxiety and restore a sense of agency. One participant, a patient experiencing long-term pain due to lumbar disc herniation, articulated this clearly:

*When I see other people saying it works, I kind of feel more confident and hopeful.*

[P6]

This statement reveals that information adoption is not always primarily about factual content. For many chronically ill patients, the process is driven by a fundamental need for emotional solace, specifically through the observation of others successfully managing similar conditions, which can be even more motivating than the informational content itself.

##### Behavioral

The behavioral layer describes the various information behaviors that patients undertake, driven by their motivations. It answers the question, “What do patients do during health information activities?” A patient’s health information behaviors can be divided into 4 interconnected and cyclical stages: information seeking, information analysis, information keeping, and information adoption.

First, information seeking encompasses both active and passive modes of access. Patients actively seek information by searching keywords, following specialist accounts, or querying platform databases. Yet a distinctive feature of the platform era is the prevalence of passive, algorithm-driven exposure, wherein content arrives without deliberate search. One participant experiencing rhinitis captured this dual dynamic:

*Because my health needs are quite specific, I can actively search for and obtain health information on my own, rather than waiting for public accounts to push content.*

[P20]

*Occasionally, when I come across or see pushed articles [about nasal congestion], I just like to click in and have a look.*

[P20]

Second, after accessing information, patients move to information analysis, in which they read, evaluate, and integrate content against their personal circumstances. This stage reveals patients as active sense-makers rather than passive recipients. One participant, a participant managing hyperlipidemia, demonstrated a rigorous approach to information analysis:

*When I encounter a professional term, such as ‘low-density lipoprotein,’ I proactively search through previously published resources and related articles until I fully grasp its meaning.*

[P5]

This behavior highlights that adoption is preceded by a deliberate process of information analysis, where patients function as active decoders of technical knowledge.

Third, information deemed valuable undergoes information keeping—a stage of micro-adoption in which patients record, save, screenshot, or memorize information for later reference. One participant explained:

*I took screenshots of what they said about ‘the three types of alcohol people with high blood pressure can't touch’.*

[P10]

This behavioral pattern reveals a layered adoption logic whereby formal adoption is typically preceded by an interim stage of informal curation, reflecting the need for patients to regulate the influx of health information and revisit it according to their own pace.

Finally, patients engage in information adoption, where curated information is applied to produce tangible health behavior changes. The progression from seeking to adoption is often iterative, involving repeated evaluation against lived circumstances. One participant, an office worker experiencing chronic lumbar muscle strain due to prolonged sitting, illustrated the seamless integration of platform-sourced health knowledge into daily life:

*During my breaks at work, I practiced the ‘seated leg raises’ I learned on Douyin; I even find time to do a few sets whenever I have a moment to rest.*

[P1]

This example highlights how platform-derived health information can be translated into embodied and routine practices, demonstrating the process through which patients integrate digital health knowledge into everyday self-management.

##### Attitudinal

The attitudinal layer encompasses patients’ subjective evaluations, experiences, and feelings toward information, answering the question, “Why do patients adopt this specific information?” Through cognitive resonance, emotional resonance, and contextual resonance, patients with chronic disease continuously perform a holistic “fit assessment” on incoming health information. This resonance framework reveals the interpretive “black box” through which patients move from encountering information to committing to its adoption.

Cognitive resonance refers to patients’ tendency to evaluate new information in light of their accumulated knowledge and lived experience. Patients with chronic disease are often inclined to adopt information that coheres with what they already understand, reflecting a comparatively deliberative mode of evaluation. For example, a participant who had studied traditional Chinese medicine for many years critically assessed online content:

*Some traditional Chinese medicine methods are completely incorrect. If people follow such approaches, it simply will not work.*

[P11]

Here, prior domain knowledge enabled the patient to reject information perceived as inconsistent with established understanding. In another instance, a participant with a lumbar disc herniation expressed greater affinity for a blogger’s advice:

*This blogger said that people with my condition can still work. A doctor once told me I had lost the ability to work, but how could that be?*

[P16]

In this case, the blogger’s message resonated more closely with the patient’s self-assessment and perceived capabilities. Across these accounts, cognitively resonant information, whether used to dismiss or to embrace particular claims, appears to carry persuasive weight. Patients actively negotiate competing sources of knowledge based on perceived alignment with their experiential understanding.

Emotional resonance refers to alignment at the affective and experiential level. Patients facing persistent pain, uncertainty, and isolation are driven not only by a need for information but also by a need for recognition, companionship, and hope. The affective framing of health information, ranging from fear and reassurance to validation, substantially determines its resonance. One participant described the anxiety generated by fear-inducing content:

*There’s a lot of information, but some of it is too scary. The title says 'Hypertension patients who don't take medication won't live for more than 5 years,' and after reading it, I felt all panicked inside.*

[P10]

This account highlights the emotional consequences of fear-inducing health information. While such content may capture attention through alarming framing, it can also generate anxiety and psychological distress among patients. In contrast, positive emotional resonance actively facilitates adoption. A thyroid cancer participant shared:

*Seeing other patients who are still healthy ten years after their surgery makes me feel less anxious.*

[P7]

The social proof embedded in peer experience functions as a powerful adoption trigger, demonstrating that the emotional community afforded by digital platforms can be a genuine resource for chronically ill patients.

Contextual resonance refers to alignment between information and the patient’s practical life circumstances, including economic resources, physical environment, family structure, and daily routines. Patients continuously evaluate information against the pragmatic constraints of their lives; information that is theoretically sound but practically unachievable fails to resonate. One participant with chronic kidney disease articulated this barrier clearly:

*The hospital says this new medicine is very effective, but it’s too expensive, and I don't have the money to buy it.*

[P8]

This account illustrates that information adoption is inextricably linked to socioeconomic factors. It underscores that “contextual resonance” is not just about understanding a message, but about the practical feasibility of acting upon it within one’s financial constraints.

### Platform Context

The platform context refers to the broader digital environment in which patients engage with health information, encompassing both the structure of information and the institutional conditions that shape how it is presented and encountered.

#### Information Context

The information context refers to the overall information structure conditions across the digital platform environment in which patients are situated. In the contemporary online ecosystem, multiple types of platforms coexist and jointly constitute the informational surroundings encountered by patients with chronic disease. These platforms differ in the information content they host, the formats through which information is presented, and the sources from which information originates. In China, this platform environment includes official health-related platforms associated with the National Health Commission, such as the Healthy China app, where authoritative health information is disseminated by governmental agencies. It also encompasses internet-based medical and health management platforms, such as Haodf.com and DXY.cn, where health information is primarily generated and exchanged by medical professionals and patients. In addition, general social media platforms, including Zhihu and Weibo, contain a substantial volume of health-related content produced and shared by ordinary users. Together, these platforms present distinct yet interconnected information structures that shape patients’ everyday health information activities.

Participants’ accounts revealed how these platform-specific information structures shaped their engagement patterns. Format emerged as a powerful determinant of engagement:

*When I'm on WeChat official accounts, I like to read articles with both pictures and text. It’s too hard to read if it’s all text.*

[P9]

This preference reflects how platform-determined content formats condition not merely aesthetic choices but patients’ actual capacity to process and engage with health information—patients with lower literacy or visual fatigue may effectively be excluded from text-heavy environments.

Content characteristics similarly influenced access. As one participant noted,

*I usually see if the title is appealing to me, and based on that, I decide whether to click on it.*

[P17]

suggesting that the way health information is presented—such as through titles—affects users’ initial engagement. At the same time, participants also emphasized the importance of the content itself. For example, one participant explained,

*I prefer reading interesting articles. Some content presents complex medical knowledge in a vivid and engaging way, which makes it more appealing to read.*

[P9]

This indicates that beyond initial attention, the intrinsic qualities of content, particularly its clarity and vividness, play a key role in attracting and retaining users’ interest. Source credibility functioned as a third decisive filter:

*I still love watching videos posted by doctors.*

[P16]

*Because I have a set of health bloggers I regularly follow, I typically stick to the information they provide.*

[P27]

This preference indicates that patients rely on source-related cues, including both professional identity and familiarity with specific content creators, when filtering health information on social media platforms.

#### Institutional Context

The Institutional Context refers to the constraints and enabling conditions embedded at the platform level, including technological infrastructures, structural characteristics, and governance mechanisms. Patients’ accounts revealed how these institutional dimensions shaped their health information adoption practices in consequential ways.

Algorithmic recommendation systems emerged as a primary mechanism through which platforms structure patients’ information exposure. Digital platforms commonly rely on recommendation algorithms to organize and prioritize information streams, determining what types of health content users are most likely to encounter. One participant described how algorithmic curation becomes the gateway for health information exposure:

*Most of the time I just watch whatever the platform recommends. If a health video appears on the homepage, I'll usually click on it and see what it says.*

[P19]

This account suggests that recommendation algorithms function not merely as neutral distribution mechanisms but as consequential filters that determine which health information enters patients’ consideration set, thereby structuring the conditions of possibility for subsequent adoption decisions.

Beyond technological mediation, the structural characteristics of platforms themselves, including institutional endorsement, ownership background, and perceived authority, shape users’ trust judgments independent of specific content evaluation. Government-sponsored platforms provide a salient example:

*Every morning I use the 'Xuexi Qiangguo' app to watch the 'Healthy China' channel. The 'Famous Doctors' Lecture Hall' segment is a must-watch for me every episode. I feel the content there is more reliable.*

[P23]

This account indicates that platform-level institutional identity functions as a credibility heuristic that precedes and conditions patients’ engagement with individual pieces of health information. The governmental affiliation establishes a baseline trust orientation that influences subsequent information adoption behaviors.

Platform governance mechanisms further shaped patients’ trust assessments through visible enforcement actions. Moderation decisions, such as account bans, content removals, or warning labels, serve as authoritative signals that influence both retrospective and prospective credibility judgments. One participant described how a platform’s intervention altered their evaluation of a previously trusted source:

*I used to watch his health videos quite often, but later the platform banned his account for spreading misinformation. After that, I felt the things he said before were probably unreliable.*

[P17]

This account reveals that governance interventions function as institutionalized trust-making mechanisms, prompting users to reassess not only future content from a source but also the trustworthiness of previously consumed information.

### Intervening Conditions

Throughout the health information adoption process, individual circumstances and environmental conditions shape how patients engage with information.

#### Individual Circumstances

At the individual level, patients’ foundational characteristics, information literacy, and illness experiences shape how they approach health information on digital platforms. At the individual level, patients’ foundational characteristics, information literacy, and illness experiences shape how they approach health information on digital platforms. Foundational characteristics, however, have been extensively examined in prior research and are therefore not elaborated on in this study.

Information literacy exercises perhaps the most pervasive moderating influence, shaping every stage of the adoption process from seeking to evaluation. A striking contrast emerges between patients with lower and higher information literacy levels. Two participants whose digital competencies differed substantially described contrasting ways of engaging with online health information:

*My literacy level isn’t good, so I usually don’t read. I watch videos and listen to audio playback.*

[P23]

The same participant also noted:

*Whatever information shows up on my phone, that’s what I watch.*

[P23]”

By contrast, another participant described a markedly different pattern of engagement:

*I generally don’t watch videos because they contain too little information and take too much time. Instead, I read a vast number of text-based articles for medical information.*

[P20]

He further explained that he prefers to search directly using specific keywords:

*I mostly search for information by entering keywords.*

[P20]

For the participant with limited literacy resources, video and audio content provide accessible entry points into health knowledge, yet they also increase reliance on algorithmically curated audiovisual feeds. In contrast, the participant with stronger digital competencies actively seeks out dense, text-based materials and engages in more extensive comparison across sources. Information literacy shapes the format of content consumed, as well as the depth of engagement, the degree of autonomy in information selection, and the evaluative strategies applied.

Illness experience constitutes a second important individual moderator. The nature and duration of a patient’s illness condition shape both the urgency and the character of their information engagement:

*I’ve had lumbar disc herniation for six or seven years. The daily pain severely affects my life, and now my social media feed is entirely filled with lumbar exercises. I particularly enjoy watching shared experiences from other bloggers who also have lumbar disc herniation.*

[P16]

Another thyroid cancer participant diagnosed 5 years ago stated:

*When first diagnosed, I scrolled through short videos every day because I was desperate to know my life expectancy. After learning that regular medication keeps my lifespan unaffected, I searched less. Now, I hardly pay attention to that information at all.*

[P7]

These accounts demonstrate that illness experience acts as a dynamic moderator, whereby the nature of the condition determines the longevity and intensity of information engagement. While chronic, symptomatic conditions can lead to persistent, algorithm-saturated seeking for peer support, the resolution of survival anxiety in other cases may result in a gradual detachment from health information as the condition becomes a stabilized part of daily life.

#### Environmental Conditions

The environmental dimension includes emergency situations, policy changes, and social relationships, all of which influence patients’ health information adoption behaviors by altering the external conditions within which adoption propensity is activated.

Policy changes exert systemic influence on the information environment itself. When government authorities introduce chronic disease management plans, promote family doctor programs, or launch public health information platforms, they restructure the channels, authoritative sources, and directions of health information supply:

*Since the community started the family doctor program, I no longer wander around various websites. I just follow the guidance pushed through the official community health platform.*

[P23]

For patients who orient their health behaviors around official guidance, policy changes can trigger wholesale shifts in the information ecosystems they inhabit—a form of top-down moderation that complements platform-level algorithmic curation.

Social relationships function simultaneously as information filters and amplifiers. The social network acts as a parallel information channel that often carries decisive trust weight:

*Whenever I find a new exercise for my back on an app, I first consult my niece who is a doctor. Only after he confirms that the movements are safe for my specific condition do I feel confident enough to actually practice them.*

[P16]

This account illustrates how interpersonal relationships can serve as a high-trust verification layer that patients consult alongside. The family member functions as a trusted information broker whose endorsement or skepticism shapes subsequent platform engagement.

Emergency situations represent a third environmental moderator that dramatically intensifies information-seeking urgency and lowers adoption thresholds. During crisis periods such as epidemic outbreaks or drug shortages, patients face compressed decision timelines and heightened anxiety:

*It’s winter now, and epidemics are more common, so I pay attention to preventive healthcare.*

[P20]

The seasonal urgency expressed here reflects a contextual trigger that temporarily amplifies the salience of health information and reconfigures the criteria for resonance. During the COVID-19 pandemic, one participant, worried about not being able to buy chronic disease medication, stated:

*I’ll try what’s said online, whether it’s right or not.*

[P28]

The emergency context dramatically lowers the critical evaluation standards that would normally govern adoption decisions, leading patients to accept information they would ordinarily verify more carefully. What this reveals about the moderating role of emergency situations is not merely heightened engagement but a qualitative shift in the epistemic standards applied.

### The Mutual Shaping Process: Platform-Patient Coconstruction

#### Overview

The platform information environment is shaped by a platform’s Information Context and Institutional Context. A patient’s information behaviors, or the behavioral layer, are embedded within this environment. Through a process of continuous iteration, these factors together form a personalized ecosystem of health information content.

#### How Platforms Shape Patients

A patient’s initial information environment on a platform is determined by underlying commercial factors, traffic allocation, algorithmic technology, platform rules, and existing users. As Suzor’s [19] research suggests, the platform dictates which information is presented and how, meaning the initial environment is essentially generated at random. Once the patient enters this environment, the platform’s institutional context, including its algorithms, interactive design, and rules constrains and guides their health information activities. One participant illustrated this mechanism of platform-induced habitual engagement:

*Because this platform offers points for daily check-ins, I go on it to browse every day.*

[P16]

Other patients described experiences of being manipulated by the platform’s algorithms.

*It’s as if the platform knows exactly what I’m worried about. Once I click on one health tip, the feed just keeps pushing similar videos. I find myself scrolling for an hour without realizing it—I just can’t stop.*

[P19]

In addition, some participants noted that platform labeling policies helped them better identify promotional health content. As one participant explained:

*Some health information is actually just promoting supplements. I couldn’t tell the difference before, but since the platform added ‘Advertisement’ labels, I now understand the true intent behind the content.*

[P17]

The gamification element embedded in the platform’s design functions not merely as an incentive but as a behavioral shaping mechanism that reliably increases daily exposure to health content, producing sustained engagement without explicit instruction. Algorithmic recommendations further structure what information patients encounter once they enter the platform. For most participants, health information engagement took a largely passive form. This systemic influence extends to the interpretive process, where platform-level markers like “Advertisement” labels provide the structural cues that guide how patients identify and categorize commercial intent.

#### How Patients Shape Platforms

Information adoption is a bidirectional interaction between the individual and their information environment. A patient’s information behaviors on a platform, in turn, actively shape the platform’s Information Context and Institutional Context, which then alter subsequent information presentation.

Regarding the Information Context, a prime example is a participant’s proactive reporting of false health information.

*Whenever I come across incorrect health information, I report it. Usually, the platform processes the report and then provides me with feedback regarding the final outcome of the review.*

[P29]

When the platform receives these reports, it deletes the noncompliant or illegal content, thereby changing the Information Context.

As for the Institutional Context, platforms use data from a patient’s information behaviors to generate a user profile. This profile then informs the recommendation algorithms, which suggest relevant information. One participant echoed this sentiment, noting:

*The more I scroll, the more the platform pushes content to me. Now, everything that appears on my phone is directly related to my condition.*

[P19]

This account illustrates the self-reinforcing logic of the feedback loop, where ongoing user interactions trigger recommendation cycles that progressively stabilize and strengthen a personalized health information ecosystem.

Conversely, some patients express frustration with the platform’s algorithmic recommendations and consciously adopt strategies to resist this influence. One participant described a deliberate counter-strategy:

*I found the platform’s recommendations were making me too anxious; a lot of them were about failed treatments. So, I clicked the 'dislike' button or repeatedly searched for successful treatment cases, which made it reduce the push of those health-related posts.*

[P7]

The patient leverages the platform’s own feedback affordances (the “dislike” function) as instruments of intentional self-curation. This constitutes a form of lay algorithmic literacy—the patient has developed a practical model of how the recommendation system operates and uses that understanding to reshape their information environment.

Whether patients accept or intentionally resist the platform’s recommendations, the final outcome is fed back into the platform’s data analysis. This allows the platform to, on one hand, use its technology and interactive design to further present a more personalized health information content ecosystem. On the other hand, it can also adjust its technology, interactive design, and community rules based on user data, achieving a cyclical and mutually constitutive shaping process.

## Discussion

### Principal Findings

This study provides 3 principal insights into how patients with chronic disease adopt health information in platform-mediated environments.

First, patients with chronic disease develop sustained adoption orientations rather than making discrete adoption decisions. Because chronic disease management requires ongoing self-care over extended periods, patients repeatedly engage with health information through iterative cycles of seeking, analyzing, keeping, and adopting. These activities are driven by multiple motivations—including disease learning, information verification, and comfort seeking—and involve continuous judgments related to cognitive, emotional, and contextual considerations. What is particularly notable is that these motivations, behaviors, and attitudes form a layered structure that evolves through accumulated experience, suggesting that adoption is better understood as a developing propensity instead of a momentary intention.

Second, platform environments actively structure the conditions under which patients encounter and evaluate health information. Patients’ information behaviors unfold within dual contexts: informational contexts characterized by diverse sources, varying quality, and multiple formats; and institutional contexts shaped by algorithmic recommendations, platform attributes, and governance mechanisms. These contexts shape what information patients are able to see, access, and consider credible, rather than merely serving as a background for their behaviors. This finding suggests that the platform environment functions as a structured and governed setting, not simply a neutral space.

Third, patients and platforms continuously shape each other through ongoing interactions. While platform algorithms influence information visibility, patients actively engage with, adapt to, and sometimes resist these algorithmic structures through practices such as selective engagement, content reporting, and cross-platform verification. These patient behaviors generate data that feed back into platform systems, influencing subsequent recommendations. Over time, these reciprocal dynamics produce personalized information ecosystems that reflect both platform logics and individual patient practices. This coconstruction process suggests that health information adoption cannot be understood by examining either patients or platforms in isolation.

### Theoretical Integration and Contribution

#### Reconceptualizing Health Information Adoption as a Dynamic Process

This study contributes to the literature on health information behavior by reconceptualizing health information adoption as a dynamic and process-oriented phenomenon. For patients with chronic disease, health information adoption emerges through continuous and iterative engagement with digital information environments. Because chronic disease management involves long-term uncertainty, symptom monitoring, and treatment adjustments, patients repeatedly search for, verify, interpret, and revisit health information over time. These activities form an ongoing cycle in which information exposure, evaluation, and behavioral responses are repeatedly revisited. Adoption thus represents an evolving orientation toward health information engagement that develops through repeated interactions with digital information environments.

This process-oriented understanding extends prior qualitative research on chronic disease patients’ information behaviors. Sun et al [5] examined how patients navigate between information avoidance and seeking, highlighting the emotional and cognitive tensions in their information practices. Building on this work, our findings show that patients’ engagement with health information is not limited to such tensions but develops through sustained and iterative interactions with digital information environments. Over time, these interactions give rise to relatively stable orientations toward health information engagement.

To conceptualize this evolving orientation, we introduce the notion of adoption propensity. Adoption propensity refers to a developing tendency toward engaging with health information, shaped through accumulated experiences, motivational drivers, and evaluative judgments. Through repeated interactions with digital health content and ongoing disease management practices, patients form patterns that influence how new information is interpreted and whether it is incorporated into their health management. In contrast to behavioral intention in dominant adoption models [7-14], adoption propensity reflects a preintentional and dynamic orientation grounded in sustained experience rather than a discrete decision point.

By foregrounding this evolving orientation, this study shifts the analytical focus of health information adoption from discrete decision-making to longitudinal engagement processes. This perspective complements existing health information adoption research by offering a process-oriented framework grounded in patients’ lived experiences that captures the dynamic nature of information engagement in contemporary digital health environments.

#### Integrating Platform Into Health Information Adoption

Beyond reconceptualizing adoption as a dynamic process, this study highlights the intertwined nature of platform architectures and health information practices. While prior research on health information behavior has predominantly focused on individual cognitive evaluations and personal literacy, our framework, grounded in participants’ lived experiences, shows that adoption is shaped through interactions with the sociotechnical and governance structures of digital platforms.

While existing platform studies have illuminated the infrastructural power of digital platforms in shaping general information visibility [21], this research advances theoretical understanding by unpacking how platforms reconfigure patients’ daily sensemaking of health information. Rather than treating platforms as neutral conduits or passive backgrounds, the emergent theoretical model captures how platforms actively participate in patients’ ongoing information practices. The grounded analysis reveals that participants continually negotiate their informational journeys through iterative interactions with curated feeds and search mechanisms. By illustrating how these algorithmic encounters shape both the emergent discovery and the sustained visibility of medical content, this study extends traditional health information behavior theories. It conceptualizes the platform as an active, vital participant in the meaning-making process, thereby offering a more situated understanding of digital health adoption.

Building on this perspective, the study further contributes to theoretical development by elucidating how platform governance mechanisms intricately weave into patients’ continuous evaluation of health information credibility. Previous literature has frequently approached platform governance primarily through a regulatory lens, emphasizing its institutional role in rule-setting and content moderation [19,23]. However, the present grounded analysis theoretically repositions phenomena such as account suspensions, community guidelines, and content removal as critical sensemaking resources for users. Within these digitally mediated environments, participants actively interpret and rely upon visible governance practices as essential cues for determining medical reliability. Consequently, this research enriches existing trust paradigms by demonstrating how institutional governance practices are intimately folded into everyday epistemological evaluations. This theoretical shift moves the analytical focus beyond isolated source-characteristic assessments, highlighting instead how users continually navigate and integrate platform governance structures to establish trust in their health information journeys.

#### A Patient–Platform Coconstruction Perspective

Building on the process-oriented understanding of health information adoption and the role of the platform, this study proposes a patient–platform coconstruction perspective to explain how health information adoption evolves in platform-mediated environments. The notion of “co-construction” draws from science and technology studies scholarship on the mutual shaping of technology and society [34], adapted here to capture the reciprocal dynamics between patients’ information practices and platform systems.

Existing research on digital platforms has emphasized the constitutive power of platforms in structuring information environments and shaping user behavior [11]. However, the findings of this study suggest that health information adoption involves not only platform influence but also ongoing patient responses that actively engage with, adapt to, and sometimes resist platform structures. Participants described various strategies for navigating platform environments, such as selectively following certain information sources, reporting misleading content, avoiding particular recommendation streams, or intentionally cross-checking information across multiple platforms. These practices resonate with van Dijck et al’s [35] observation that users are more than passive subjects of platform power and develop “tactics” to negotiate platform logics. Our findings extend this insight by revealing specific tactics patients with chronic disease use in health information contexts—including what some participants described as “training” the algorithm by deliberately engaging with or avoiding certain content types.

At the same time, patients’ repeated interactions with health information generate behavioral data that feed into platform algorithms and influence subsequent recommendation patterns. Over time, these interactions create feedback loops in which patients’ information practices shape the informational environments they encounter, while platform systems simultaneously structure the visibility and accessibility of health information. This reciprocal dynamic aligns with Gillespie’s [36] concept of “calculated publics,” wherein algorithmic systems and user behaviors mutually constitute the information environments users experience. In the context of chronic disease management, where patients engage with health information over extended periods, these reciprocal dynamics become particularly pronounced, resulting in highly personalized information ecosystems that reflect both platform logics and individual patient practices.

By highlighting these ongoing interactions, the patient–platform coconstruction perspective provides a relational understanding of health information adoption in digital health environments. This perspective contributes to platform studies by providing micro-level empirical evidence of how users and platforms mutually constitute digital information ecosystems in health contexts, while also extending health information behavior research by foregrounding the role of platform structures in shaping adoption processes.

### Practical Implications

The findings of this study, while derived from a specific sample of patients with chronic disease in urban China, offer several insights that may inform practice in platform-mediated health information environments.

For platform designers and operators, the finding that patients develop sustained engagement patterns with algorithmically recommended content suggests that platform design might benefit from balancing engagement metrics with health-oriented goals. Mechanisms that promote exposure to diverse authoritative sources, rather than continuously reinforcing existing preferences, may help address the information concentration patterns observed in this study. The finding that platform governance actions influence patients’ credibility assessments has implications for content moderation practices. When platforms remove health-related content or suspend accounts, these actions function not only as regulatory mechanisms but also as trust signals that patients interpret when evaluating information sources. This suggests the value of transparent and consistent moderation practices, particularly for health-related content where credibility assessment is consequential. Additionally, the observed differences in how patients with varying information literacy levels navigate platform environments point to the potential value of literacy support features, such as contextual prompts or credibility indicators, that assist users in developing critical evaluation skills.

For health care providers and public health agencies, the finding that patients frequently bring platform-mediated information into clinical encounters suggests that constructive engagement with such information may support trust-building and shared decision-making in clinical practice. The stable platform use patterns observed among participants indicate that digital platforms may serve as viable channels for reaching chronic disease populations in public health communication. However, the diversity of platform preferences across participants also suggests that multi-platform strategies may be necessary to ensure broad reach, particularly in contexts where patients access health information across government-affiliated platforms, professional health sites, and general social media such as WeChat and Douyin. The intensive information seeking and emotional vulnerability described by newly diagnosed patients point to a critical transition period during which supportive resources and guided information pathways may be particularly valuable in helping patients develop sustainable information practices.

For policymakers and regulators, the coconstruction dynamics revealed in this study—wherein patients’ behaviors and platform algorithms continuously shape each other over extended periods—raise questions about responsibility and accountability in platform-mediated health information environments. As patients’ long-term information exposure patterns are shaped by algorithmic systems, regulatory frameworks might consider how to ensure that platform governance mechanisms support rather than undermine health information quality and accessibility. The finding that platform moderation decisions function as credibility signals in patients’ evaluations suggests that regulatory oversight of content moderation practices may be warranted, particularly for health-related content where misinformation can have serious consequences. Policies that establish standards for transparency and consistency in platform governance of health information may help protect public trust while respecting platform autonomy.

For patients and patient advocacy groups, the finding that some patients actively shape their information environments through selective engagement and cross-platform verification demonstrates patient agency within algorithmically mediated spaces. Patient education programs might benefit from explicitly addressing platform literacy, including understanding algorithmic logic and intentional feature usage, to support informed navigation of digital health information environments. The role of family members and patient communities in influencing information adoption decisions points to the potential for patient communities to function not only as emotional support networks but also as collective information verification networks, where members collaboratively evaluate and share reliable health content.

### Limitations and Future Work

First, this study was conducted among urban patients with chronic disease in Chengdu, China, within the context of Chinese digital platforms such as WeChat and Douyin. Differences in platform architectures, governance mechanisms, and broader digital infrastructures may limit the transferability of the findings to rural settings or to other cultural and platform environments. Future research could examine health information adoption across diverse sociotechnical contexts, including rural populations and non-Chinese platform ecosystems, and conduct cross-platform or cross-cultural comparative studies to assess the generalizability and contextual sensitivity of the proposed model.

Second, this study relied on participants’ self-reported and retrospective accounts of their health information behaviors. While this approach is well-suited to exploring subjective meanings, interpretations, and sense-making processes, it may be subject to recall bias and social desirability bias. Future research could adopt longitudinal and mixed method designs, such as diary studies, in situ interviews, or digital trace analysis, to capture real-time interactions between patients and platforms and to examine how information adoption behaviors and platform influences evolve over the illness trajectory.

Third, the theoretical model proposed in this study was developed through qualitative grounded theory and is intended for theory generation rather than hypothesis testing. As such, causal relationships among model components cannot be empirically established. Future quantitative research could operationalize key constructs identified in this study and test their relationships with health information adoption outcomes. In addition, intervention-based studies, including randomized controlled trials, could evaluate whether leveraging specific model components can improve health information adoption and patient outcomes. Relevant components include algorithmic literacy training, curated authoritative information feeds, and peer-moderated information environments.

### Conclusions

This study reframes patients with chronic disease’s digital health information adoption as a sociotechnical process shaped by the reciprocal interaction between patients and platform environments, rather than a purely individual and rational decision-making behavior. By highlighting the governing roles of algorithms, platform structures, and institutional mechanisms, the proposed model extends existing health information adoption theories to better capture platform-era dynamics. The findings underscore that effective patient empowerment and information quality improvement cannot rely solely on individual literacy but require ethically designed platforms, responsible algorithmic governance, and coordinated policy interventions. More broadly, as digital platforms increasingly function as sites of health governance, understanding and shaping platform–patient relationships will be critical for mitigating misinformation risks, reducing health inequities, and improving long-term health outcomes.

## Supplementary material

10.2196/85229 Checklist 1 SRQR checklist.

## Abbreviations

SRQR: Standards for Reporting Qualitative Research
